# Recent Advances in the Development and Efficacy of Anti-Cancer Vaccines—A Narrative Review

**DOI:** 10.3390/vaccines13030237

**Published:** 2025-02-25

**Authors:** Kajetan Kiełbowski, Paulina Plewa, Jan Zadworny, Estera Bakinowska, Rafał Becht, Andrzej Pawlik

**Affiliations:** 1 Department of Physiology, Pomeranian Medical University, 70-111 Szczecin, Poland; paulina.plewa@op.pl (P.P.); j.r.zadworny@onet.pl (J.Z.); esterabakinowska@gmail.com (E.B.); 2 Department of Clinical Oncology, Chemotherapy and Cancer Immunotherapy, Pomeranian Medical University in Szczecin, 71-252 Szczecin, Poland; rafal.becht@pum.edu.pl

**Keywords:** cancer immunotherapy, anticancer vaccines, nucleic acid-based vaccines, personalized oncology, oncology

## Abstract

Immunotherapy is an established and efficient treatment strategy for a variety of malignancies. It aims to boost the anticancer properties of one’s own immune system. Several immunotherapeutic options are available, but immune checkpoint blockers represent the most widely known and investigated. Anticancer vaccines represent an evolving area of immunotherapy that stimulate antigen-presenting cells, cytotoxic responses of CD8+ T cells, and the presence of memory T cells, among others. Over the years, different approaches for anticancer vaccines have been studied, such as mRNA and DNA vaccines, together with dendritic cell- and viral vector-based vaccines. Recently, an accumulating number of clinical studies have been performed to analyze the safety and potential efficacy of these agents. The aim of this review is to summarize recent advances regarding different types of therapeutic anticancer vaccines. Furthermore, it will discuss how recent advances in preclinical models can enhance clinical outcomes.

## 1. Introduction

Cancer immunotherapy is centered around the idea of harnessing the potential of the immune system to fight cancer. Along with conventional methods, such as surgery, chemotherapy, and radiotherapy, it has emerged as another pillar of oncological treatment, providing a complementary approach to previously non-curable tumors [1,2]. So far, most of the approved cancer immunotherapies make use of monoclonal antibodies [3], but anticancer vaccines continue to draw more and more attention.

**Figure 1:**
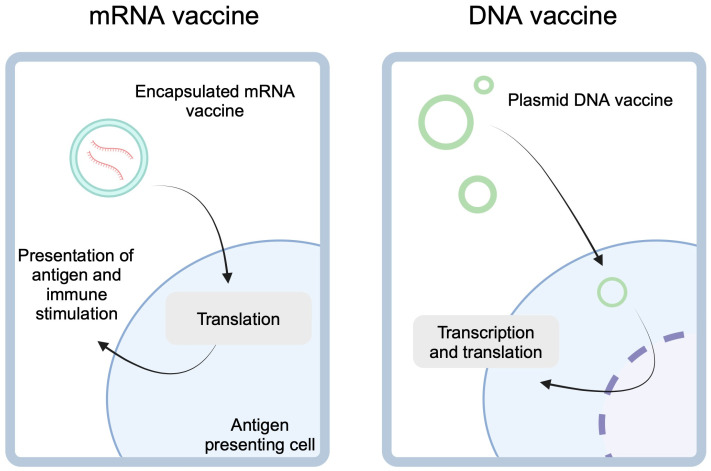
mRNA-based cancer vaccines utilize lipid encapsulation to enter the antigen-presenting cells. After the release of the cargo, mRNA is translated into peptides which can be presented to stimulate immune responses targeted at presented antigens. DNA vaccines, on the other hand, are gene-expressing plasmids. These molecules need to enter nucleus for transcription before they can undergo the translation. Created in BioRender. Kiełbowski, K. (2025) https://BioRender.com/f25r737.

Anticancer vaccines can have preventive and therapeutic character. The former type aims to reduce the risk of developing cancer. Vaccines against human papilloma virus (HPV) and hepatitis B virus (HBV) represent pivotal anticancer preventive vaccines. Therapeutic vaccines promote anticancer properties of immune cells, thus enhancing their cytotoxic responses. By contrast to the previous group, therapeutic vaccines aim to eliminate already existing cancer. Although the history of the latter group dates back to the late 19th century and the pioneering works of William B. Coley, therapeutic anticancer vaccines have only recently experienced a true renaissance [4,5]. Over the years, several types of therapeutic cancer vaccines have been developed. These include nucleic acid-based vaccines (NAVs) such as mRNA or DNA vaccines (Figure 1). The former is mainly designed using tumor-associated antigens (TAA) and tumor-specific antigens (TSA) [6]. mRNA vaccines can encode up to several antigens at the same time, thanks to which a large number of epitopes are presented. Due to the possibility of mRNA production using in vitro transcription, direct translation occurs immediately after entering the cytoplasm. This delivery method reduces the risk of integration into the patient’s genome [7]. DNA vaccines involve gene-containing plasmids, which need to undergo both transcription and translation. Other anticancer vaccination mechanisms include utilizing neoantigens, viruses, and dendritic cells (DCs). In this review, we explore recent advances in the development and clinical implementation of anticancer vaccines. We discuss novel approaches to inducing antitumor immunity along with the underlying molecular mechanisms.

## 2. Therapeutic Cancer Vaccines

### 2.1. mRNA-Based Cancer Vaccines

There are several methods of delivering mRNA vaccines. One of them is the so-called “naked” mRNA vaccine, which involves a direct injection of mRNA in a buffer, without a carrier. To increase mRNA stability and prevent damage, nucleic acid can be delivered through lipoplexes or lipid nanoparticles. The mRNA molecule that is foreign to an individual is immunogenic, thus activating innate immunity. The use of lipid-based encapsulation techniques allows them to stabilize the molecule and play a role in uptake by antigen-presenting cells, where the cargo is released [8]. Moreover, mRNA-based vaccines can be introduced into the human body with the help of adjuvants, which are designed to increase the immunogenicity of the preparation. In addition, this process can be mediated by encapsulation, which increases the stability of naked mRNA [9]. The vaccine can be introduced intravenously, intramuscularly, intradermally, subcutaneously, or directly into the tumor [10]. Another method to increase the immunogenicity of an mRNA vaccine was recently proposed by Guo and colleagues [11]. Researchers utilized different cellular compartments to significantly increase mRNA expression and enhance immune responses. The authors prepared nanostructures that would assemble in lysosomes. Escaping these structures into the cytoplasm would promote translation, thus effectively stimulating antigen expression.

TAAs are classified as autoantigens, which are found within cancer and non-cancer cells. In the former, these antigens are characterized by different post-translational modifications and are expressed at relatively higher levels. Therefore, the human immune system can identify them as items of interest to the respective immune mechanisms [12]. There are three categories of TAA. Widespread and overexpressed antigens are associated with tumor suppressor proteins and antiapoptotic proteins, i.e., p53, livin, surviving, and HER-2/neu. Another category is differentiating antigens, which are demonstrated selectively by the cell line from which cancer cells developed. The most well known are antigens of melanocytes—gp100 (glycoprotein 100) and Melan-A/MART-1—as well as prostate antigens, such as prostate-specific antigen (PSA) [6,12,13]. The last category includes antigens referred to as cancer testis antigens (CTA) testicular cancer antigens. They are expressed in the so-called immune-privileged tissues, thanks to which defective expression in cancer cells makes them highly immunogenic. Interestingly, CTAs are not expressed on HLA-I molecules, so they are not presented to lymphocytes [12]. The vaccines targeting TAA have not lived up to their expectations. Firstly, they are characterized by reduced effectiveness due to their relatively low tumor specificity, because they also occur within non-tumor cells. Additionally, they must break tolerance mechanisms, thus influencing the size of the T-cell response. Many of the agents are characterized by a weak and insufficient immune response. On the other hand, such vaccines may induce autoimmunity [12,14].

**Figure 2:**
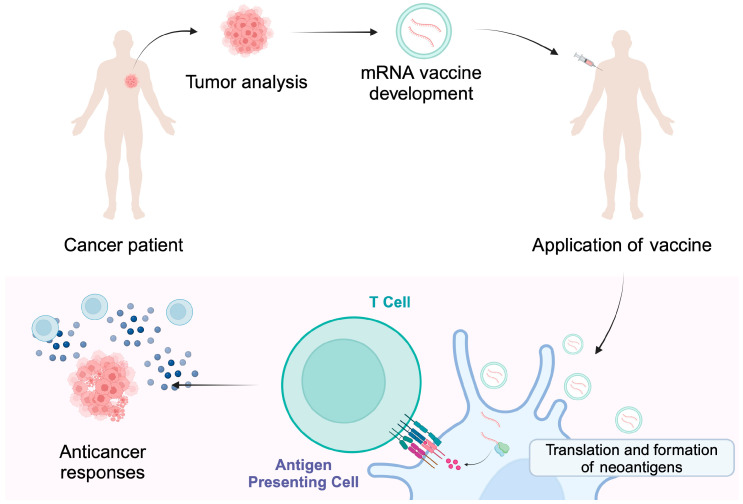
Individualized cancer vaccines are developed based on the tumor mutational status and the presence of neoantigens. Encapsulated mRNA molecules can enter antigen-presenting cells where a release of cargo occurs. The translation and presentation of neoantigens stimulate immune responses that are targeted at cancer cells, thus providing cytotoxic activity. Created in BioRender. Kiełbowski, K. (2025) https://BioRender.com/i22w075.

TSA, also referred to as neoantigens, are only expressed in cancer cells. They are associated with the occurrence of genetic and molecular changes occurring strictly in cancer cells [12,13]. These can be somatic mutations arising in coding and non-coding regions, including long non-coding RNAs, protein-coding gene untranslated regions, pseudogenes and antisense strands, and unconventional DNA reading frames [6]. They can also arise through chromosome rearrangements and the expression of viral oncogenes [12]. All genetic changes are unique to each person and trigger a characteristic anticancer response. Therefore, it is possible to use targeted therapy while maintaining healthy tissues [15]. The process involves the excision of a specific tumor and then the identification of specific antigens through next-generation sequencing. The next step is the introduction of antigens by injection into the patient in order to trigger an appropriate immune response that results in the tumor being attacked [16] (Figure 2). Compared to TAA, TSA has a much higher affinity for major histocompatibility complex (MHC) and T-cell receptor (TCR) [13,17].

Several studies described the potential use of mRNA-based anticancer vaccines. Preclinical in vitro studies, in vivo experiments, together with clinical trials have been already published that described the uses of these vaccines. Moreover, researchers are exploring the potential of combination mRNA vaccines with immunotherapeutics, as well as various modifications of vaccines to further increase their efficacy.

The mRNA4157 (V940) vaccine is an individualized vaccine that encodes up to 34 neoantigens. The agent is developed to match human leukocyte antigen types and the mutational character of the tumor. Its efficacy has been examined clinically. Major findings were recently published regarding melanoma, a type of cancer in which immunotherapy holds a key role. KEYNOTE-942 [18] phase 2b trial represents one of the largest and most promising recently published clinical trial results. The included cohort was composed of patients with completely resected high-risk melanoma (stages IIIB-IV). These patients received adjuvant therapy composed of either pembrolizumab, one of the recommended adjuvant immunotherapeutics [19], or pembrolizumab combined with the mRNA4157 vaccine. The recurrence-free survival (RFS) at 18 months for the study and the control groups were 79% and 62%, respectively. The trial also revealed the manageable safety profile of the combination [18]. Thus, KEYNOTE-942 proved that using anticancer vaccine as an adjuvant treatment in patients with melanoma reduces the risk of recurrence. INTerpath-001 is a phase III clinical trial that will examine the mRNA4157 vaccine with pembrolizumab in the cohort of patients with stage II-IV resected melanoma. The trial is expected to include over one thousand participants and allocate them into the study and control groups [20]. Interestingly, a recent trial demonstrated benefits of using pembrolizumab in patients with stages IIIB/D-IV melanoma in neoadjuvant and adjuvant settings [21]. It is an open question whether the combination of pembrolizumab with mRNA4157 would also provide benefits when used before the surgery. KEYNOTE-603 study is another trial that investigated the V940 vaccine in resected non-small cell lung cancer (NSCLC) and melanoma. This phase I study demonstrated enhanced T cell responses to novel antigens [22]. Several clinical trials are currently being performed to further investigate the safety and efficacy of V940 (NCT05933577 [23]; NCT06623422 [24]; NCT06295809 [25]; NCT06307431 [26]; and NCT06077760 [27]). Among these studies, the vaccine will be examined in neoadjuvant settings in patients with cutaneous squamous cell carcinoma [25].

Melanoma-associated antigen A3 (MAGE-A3) is a TAA that is expressed in approximately 75% of adult melanoma patients [28]. In a cohort of adults with melanoma, MAGE-A3 expression was positivity associated with worse event-free survival, as compared to those not expressing tumors [28]. Although the phase 3 clinical trial did not support the use of MAGE-A3 immunotherapeutic in patients with melanoma [29], MAGE-A3 is expressed in other cancers as well. The MAGE-A3-based mRNA vaccine has been recently investigated by Choi et al. [30]. Researchers demonstrated that MAGE-A3 mRNA in lipid nanoparticles introduced to mice colorectal cancer models significantly improved survival.

mRNA-4359 is another anticancer mRNA-based vaccine engineered by the same developer as V940. This agent is far less investigated and has a different mechanism of action. It encodes immunogenic peptides of PD-L1 and indoleamine 2,3-dioxygenase 1 (IDO1) peptides. Therefore, by enhancing immune responses at cells expressing these peptides, such as cancer cells, the vaccine does not induce individualized responses, but rather global anticancer activity. Results of the phase I/II clinical trial (NCT05533697) that examined the activity of dose-escalating mRNA-4359 monotherapy in patients with advanced solid tumors who were refractory to at least one prior treatment line were presented at the 2024 ESMO Congress. Among the 19 included patients, 50% achieved a stable disease. T cell responses targeted towards the encoded proteins were observed in 93% of patients. The most common grade 1–2 AEs included fatigue, injection site pain, and pyrexia, while no dose-limiting toxicities were observed [31].

Another type of neoplasm that has been suggested to benefit from mRNA vaccines is pancreatic cancer [32]. Recent studies analyze potential genes that could be implicated in antitumor response and involved in the development of vaccines. For example, Yan and Wang [33] demonstrated ferroptosis-related genes that could become targets for a mRNA vaccine.

Autogene-cevumeran is a system that utilizes the mRNA of neoantigens and lipoplexes. It offers an individualized therapy approach to target up to 20 neoantigens. Rojas et al. [34] investigated the sequential treatment of patients with pancreatic ductal adenocarcinoma with atezolizumab, autogene-cevumeran, and mFOLFIRINOX. Monitoring 16 patients with a median of 18 months of follow-up, a median RFS and overall survival (OS) were not reached. Eight patients were considered treatment responders. In those 8 patients, researchers observed T cell clonal expansion that was vaccine related. The same observation was made for one patient in the non-responder’s cohort. Several ongoing trials aim to examine potential benefits of autogene-cevumeran in patients with cancers (NCT03289962 [35]; NCT03815058 [36]; NCT06534983 [37]; and NCT05968326 [38]).

Fan and colleagues [39] prepared a SmartNeo platform that plays an important role in cancer vaccine development. It performs HLA typing, identifies and analyzes tumor mutations and can quantify gene expression. Researchers utilized this platform to create the lypopolyplex-mRNA vaccine. The application of the agent to animal models demonstrated enhanced immune response, with increased maturation of CD103+ and CD8+ DCs, a subtype of DCs associated with antitumor activity [40]. The vaccine showed promising in vivo efficacy in colorectal cancer xenografts [39]. Thus, the abovementioned studies describe the use of advanced platforms that can create cancer specific vaccines and induce anticancer responses. Recent papers also demonstrate mechanisms to improve the efficacy of mRNA vaccines. For instance, the implementation of all-trans-retinoic acid into the mRNA vaccine was proven to increase the expression of gut-tropism molecules in T cells, therefore increasing gut homing and infiltration of immune cells into colorectal cancer in mice models [41]. Another approach involves an application or an oral mRNA vaccine that protects the compound from pH alterations and directly accesses immune cells in the intestines. This modification also prevents accumulation in the liver [42]. The use of adjuvants aims to increase immunological response to mRNA vaccine. Meulewaeter et al. described the use of glycolipid alpha-galactosylceramide (αGC) in liposomes that were in a complex with mRNA molecules. Administration of adjuvanted vaccine into mice models was associated with a significantly increased production of cytokines, including IFN, TNF, IL-2, IL-22, IL6, MCP-1, IL-4, and IL-17A. In melanoma animal models, the use of the vaccine with the adjuvant demonstrated promising efficacy [43].

When discussing the immunoregulatory properties of vaccines, one should address the issue with “cold” and “hot” tumors. The former are associated with immunosuppressive conditions, which prevent the immune system from inducing cytotoxic responses. mRNA vaccines are thought to overcome immunosuppression and enhance the pro-inflammatory tumor microenvironment. Recently, Fournier et al. [44] described the use of innovative lipid nanostructures to encapsulate mRNA. Researchers observed that the use of mRNA that expresses ovalbumin in nanostructures was able to stimulate pro-inflammatory responses of DCs by interacting with toll-like receptors (TLRs), major receptors involved in innate immunity. In ovalbumin-overexpressing animal cancer models, vaccination could suppress tumor growth and promote infiltration of tumors with immune cells, as well as increased presence of cytotoxic molecules, such as interferon, the tumor necrosis factor, and granzyme B.

### 2.2. DNA-Based Cancer Vaccines

DNA-based vaccines serve as genetic information directed against tumor antigens, which, when administered to a patient, trigger a specific immune response [45]. They can be administered in a variety of ways, such as electroporation (a physical method involving the use of direct current, which creates momentary pores within the cell membrane, allowing the vaccine to be introduced into the cell), sonoporation (the use of sound waves that allow them to penetrate the cell the outer layer of the cell), and DNA tattooing with a gene gun [46]. DNA vaccines can simultaneously deliver a multitude of antigens in a single drug [47]. They are characterized by significant antigenic specificity, safety, and have fewer side effects compared to other non-targeted therapies [48].

There are several types of DNA vaccines that are associated with the concept of increasing immunogenicity. To begin with, there are DNA vaccines based on MUC1 (mucin-1). MUC1 in humans occurs in the form of a transmembrane protein. Considering the structure, this protein is divided into two parts—the N- and C-terminal fragments—which are connected by non-covalent bonds. The first fragment is located on the surface of the membrane and consists of a signal peptide along with a VNTR region (variable number of tandem repeats). Both the VNTR region and the MUC1 protein are enriched with serine and threonine residues so that O-coupled glycosylation occurs there. In contrast, the C-terminal fragment consists of an extracellular domain, a transmembrane domain, and an intracellular domain, the latter two of which are highly conserved [49,50]. Physiologically, this protein is expressed in many organs, e.g., kidneys, lungs, pancreas, breasts, and is responsible for the formation of appropriate barriers protecting epithelial tissues and is indirectly related to signal transduction [50]. In the pathological state, i.e., in cancer, impaired glycosylation or hypoglycosylation occurs [51]. Such a vaccine constructed in the form of a recombinant eukaryotic vector was tested in mice models. After intramuscular administration, T and B cells were stimulated. First, MUC1 is degraded to peptides consisting of 8 to 12 amino acids. In the next stage, they are presented to MHC-I molecules, which stimulate CD8+ T cells, leading to CTL stimulation. MUC1 can also be released into the extracellular space, which is then recognized by APCs, leading to endocytosis and presentation to MHC-II molecules. This leads to the stimulation of CD4+ T cells, which affect differentiation into Th1 and Th2 cells. Additionally, MUC1 secreted into the extracellular space binds directly to the BCR receptor of B cells, leading to the production of antibodies [50]. Unfortunately, DNA vaccines based solely on MUC1 do not show sufficient effectiveness, which is why various adjuvants and epitopes containing T and B cells are additionally used [52].

Another method of boosting the immunogenicity of DNA-based vaccines makes use of xenogeneic DNA, which is substantially homologous to self-ortholog but encodes proteins or peptides derived from another species [53]. Thanks to the difference between autologous and xenogeneic DNA sequences, chimeric vaccines created in this way tend to trigger a stronger immune response. Unfortunately, their anticancer potential is limited by the low affinity of produced antibodies for autologous TAAs [51]. One answer to this problem is the utilization of plasmids that encode hybrid proteins resulting from the fusion of homologous and xenogeneic domains. Indeed, such constructs combine the advantages of classic DNA-based and chimeric vaccines and are thus able to induce a more versatile immune response [46].

DNA vaccines have many advantages that bring them closer to approval for use in cancer therapy. Each compound is characterized by a properly selected composition that guarantees long-term protective ability. In addition, it is safe to use, exhibits significant stability at room temperature, and has a favorable solubility profile [54]. Unfortunately, vaccines have several drawbacks, one of which is a too-low immunogenicity generation, and thus insufficient immunostimulation and DNA transfection. The dependence of the latter phenomenon is related to the structural complexity of cell and nuclear membranes in patients [10].

Currently, much effort is put into designing appropriate DNA vaccines and delivery methods, as well as investigating their clinical potential. pTVG-hp is a plasmid DNA vaccine encoding PAP, which is investigated in patients with prostate cancer. The first investigations began in 2005 but, recently, more studies have published their results. In the small-sample study summarizing the long-term outcomes of pTVG-hp vaccination in patients with non-metastatic prostate cancer, OS in castration-sensitive patients was 12.3 years, while it was 4.5 years in the castration-resistant cohort [55]. In a phase 2 trial, vaccination with pTVG-hp in non-metastatic castration-sensitive prostate cancer patients did not show significant difference in 2-year metastasis-free survival (MFS) [56]. However, a combination of the vaccine with nivolumab or pembrolizumab was suggested to prolong time to disease progression [57,58]. Two clinical trials investigating pTVG-hp with the status: “Active, not recruiting” are currently listed on clinicaltrials.gov. The NCT03600350 trial will examine vaccination with nivolumab in patients with non-metastatic prostate cancer. The NCT04090528 aims to examine the potential of combining two DNA vaccines with pembrolizumab. Specifically, pTVG-hp will be combined with pTVG-AR. The latter agent encodes androgen receptor ligand-binding domain. It was also examined clinically in patients with prostate cancer. The trial showed that the vaccine is safe and induced immune responses [59]. Similarly to pTVG-hp, the combination of the pTVG-AR vaccination could demonstrate important clinical benefits. Muralidhar et al. showed that vaccination with pTVG-AR before androgen deprivation therapy enhanced antitumor effects in murine models [60].

Recently, the results of a single-arm phase I clinical trial investigating the ERBB2 plasmid DNA vaccine in breast cancer patients were reported. Specifically, 66 stage III and IV ERBB2 (HER2)-positive breast cancer patients that completed standard therapy were included in the trial. Importantly, the results showed that the vaccine is a safe, with the most common adverse events (AEs) being injection site reaction (82%), fatigue (36%), and flulike syndrome (33%). The vaccination was associated with greater presence of central memory T cells, with differences observed depending on the dose of vaccine [61].

We have previously mentioned the unsuccessful clinical development of an mRNA vaccine encoding MAGE-A3 in melanoma. Recently Qin et al. [62] suggested that these observations could result from the activity of other MAGE antigens. With similar activity between these antigens, targeting one isoform might not be enough to suppress cancer growth. The authors recently developed a DNA vaccine that encoded multiple MAGE antigens (A2, A3, A10) and tested it in preclinical animal models. In animals harboring gemcitabine resistant pancreatic cancer, the vaccine significantly reduced tumor growth [62]. Therefore, vaccines targeting multiple MAGE antigens should be further explored. Furthermore, researchers did not observe significant differences in tumor growth in cancer that were sensitive to gemcitabine. Thus, it raises the question of whether these vaccines could be implemented in tumors resistant to particular therapies, giving further opportunities for personalized treatment. For instance, anticancer therapeutic vaccines are frequently studied in combination with immunotherapy. Yarchoan and colleagues [63] studied the efficacy of DNA plasmid-based vaccine in HCC, a malignancy which is considered less responsive to immunotherapy than other types of cancers. Researchers examined the GNOS-PV02 vaccine, a DNA plasmid that encodes up to 40 neoantigens. Moreover, the vaccine also involves a second plasmid encoding IL-12. Utilization of IL-12 aims to increase the local cellular response to antigens. The authors examined the use of vaccine in combination with pembrolizumab in a cohort of advanced HCC patients with prior treatment of multi-tyrosine kinase inhibitors. Although the small samples size must be acknowledged, the ORR (RECIST 1.1) was 30.6%, which demonstrates a promising response rate compared to the previous clinical trials [64]. Moreover, the authors describe a patient with a primarily unresectable tumor who received 5 cycles of the vaccine. Subsequently, the lesion became resectable, which demonstrates a promising response observed in HCC [63].

### 2.3. Neoantigen Vaccines

In recent years, neoantigens have been gaining more interest due to the lack of risk of immune tolerance as the human immune system recognizes these antigens as foreign. In addition, it is possible to target tumors more accurately. Neoantigens develop due to mutations and molecular alterations occurring in cancer cells. Accumulating studies have been describing the clinical use of neoantigen vaccines. For instance, NEO-PV-01 is a personalized vaccine that has been examined clinically in combination with anti-PD-1 therapeutic. The vaccines were prepared based on analyses performed on tumor samples obtained from the patients. In the NCT02897765 trial, only patients with NSCLC, melanoma, and bladder cancer with more than 50 gene fusions or point mutations were included in the vaccine design step. Importantly, the trial showed that the treatment strategy was safe. Additionally, the vaccine was found to induce immune response targeted at some of the vaccinated peptides. In vitro experiments confirmed that stimulated T cells were able to recognize and induce anticancer effects [65]. Subsequently, the NEO-PV-01 vaccine was combined with carboplatin, pemetrexed, and pembrolizumab as a first-line treatment of metastatic non-squamous NSCLC. In the study by Awad et al. [66], researchers described the vaccination of 21 patients. The authors state that the combination regiment was well tolerated, while the injection site reaction was the only AE occurring more commonly in the cohort of vaccinated participants. Despite combination strategies involving immunotherapy, researchers also describe the use of neoantigen vaccines in monotherapy [67] or with radiotherapy [68].

### 2.4. Viral Vector-Based Cancer Vaccines

Viral vector-based vaccines are used to stimulate a strong and long-term immune response. Genetically modified viruses such as adenoviruses, parvoviruses, and vaccinia are mainly used [69]. Each vector known to us today exhibits diverse traits related to affinity for a particular host cell type, the amount of genetic material delivery, and immunogenicity, all of which play a very important role in determining utility for specific cancer vaccines. This is an interesting strategy in the treatment of cancer because of the use of the natural ability of viruses to penetrate host cells and stimulate an immune response against the introduced antigens [70]. They are recognized by APCs, which consequently activate CD4 and CD8 T cells. The use of this type of vaccine results in increased immunogenicity compared to other available compounds. This is related to the presence of a pro-inflammatory environment, which is formed as a result of the expression of viral proteins. The viral adjuvant capacity inducing high immunogenicity is due to pathogen-associated molecular patterns (PAMPs) and the triggering of the innate immune response [69].

**Figure 3:**
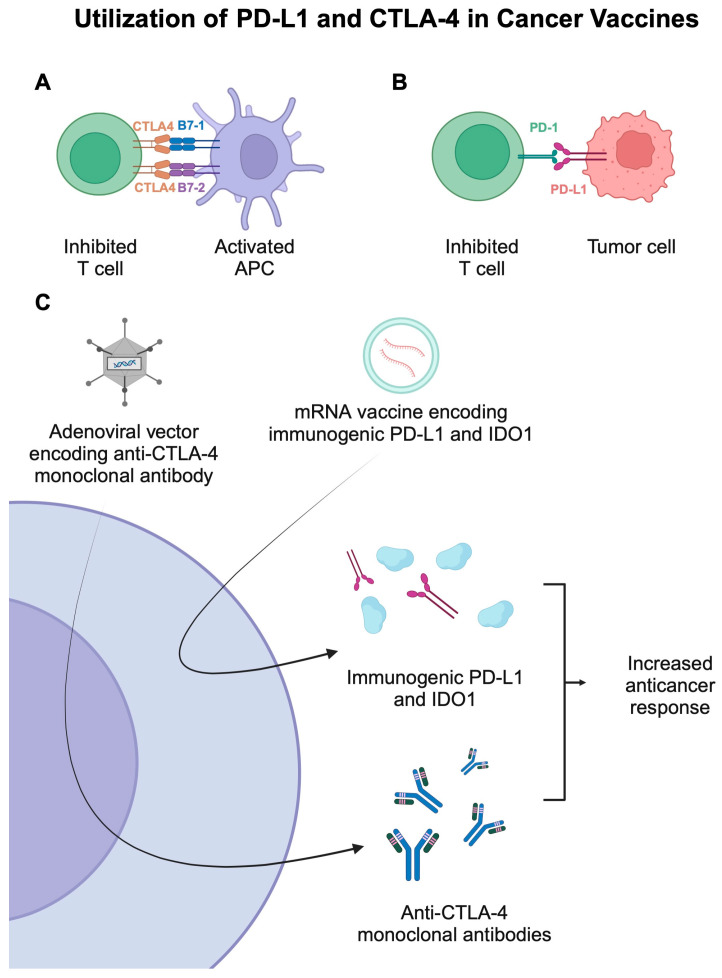
(**A**,**B**) Representation of mechanisms that suppress the activity of T cells. (**C**) The use of mRNA-based and adenovirus-based cancer vaccines that utilize immunogenic PD-L1 and mouse anti-CTLA-4 monoclonal antibodies. Created in BioRender. Kie&ðwski, K. (2025) https://BioRender.com/h84t864.

Most adenoviruses cause common upper respiratory tract infections in humans. They are enveloped, double-stranded DNA viruses. Their genome ranges from 26 to 45 kb, and the amount of genetic information carried in the form of cDNA is estimated at 7.5 kbp. An important feature of this vector is the low risk of insertion mutagenesis [69,71]. During the construction of adenovirus vectors, the E1 region is removed, completely limiting replication. By reducing pathogenicity, it is possible to focus on eliciting both humoral and cellular responses to the transgene. The lack of replication benefits security profiles when designing constructs. In addition, a region of E3 that is not needed during replication may be deleted but may nevertheless increase the ability to insert the transgene [72]. Adenoviruses are one of the most commonly used viruses in vaccine preparations thanks to their extensive tropism for many different cells. They are characterized by the ease of infection of dividing and non-dividing cells [73]. This type of virus also has many other features in their favor, i.e., the inability to integrate into the human genome, high immunogenicity, ease of transgene insertion, a simple structure, and an uncomplicated vector construction scheme. Ad5 (human adenovirus serotype 5) is the most frequently used vector, and the designed vaccines are characterized by an increased response by T cells [74]. Unfortunately, due to the prevalence of Ad5 neutralizing antibodies among the community, the effectiveness of vaccine treatment is decreasing [74]. Therefore, other ways of designing vaccines are being developed using unique serotypes, e.g., Ad26, or non-human serotypes, e.g., ChAdOx1 (chimpanzee adenovirus) [75]. ChAd68 and Gad20 (gorilla adenovirus) may prove to be very helpful as they have been confirmed to be able to trigger an immune response and stimulate T cells [76]. Several recent studies described the use of adenovirus-based vaccines in cancer patients. For instance, the PrCa VBIR is a vaccine-based therapy developed for patients with prostate cancer [77]. The treatment strategy involves the use of AdC68 vector-encoding PSA, prostate-specific membrane antigen (PSMA), and prostate stem cell antigen (PSCA), as well as tremelimumab, a monoclonal antibody targeting an anti-cytotoxicT lymphocyte- associated antigen 4 (CTLA-4), or sasanlimab, a programmed cell death-1 (PD-1) monoclonal antibody. In the phase 1 trial, treatment-emergent AEs (TEAEs) occurred in 91.1% of patients, while serious events were observed in 19.6% of metastatic castration resistant prostate cancer (mCRPC) [77]. Fatigue, nausea, and diarrhea were the most common AEs, with the respective occurrence rates of 35.7%, 21.4%, and 21.4%. Despite manageable toxicities, modest anticancer efficacy resulted in cessation of further development [77]. This clinical trial is another example of the difficulties in translating beneficial preclinical observations into clinically beneficial treatment methods. The use of an adjuvant has also been explored in the context of adenoviral vaccines. D’Alise and colleagues [78] reported the benefits of using Ad-9D9, an adenovirus loaded with a mouse anti-CTLA4 gene. The use of Ad-9D9, together with the neoantigen vaccine and anti-PD-1 therapy, significantly reduced tumor growth in colon adenocarcinoma animal models. Figure 3 illustrates the involvement of PD-L1 and CTLA-4, typical immunotherapy targets, in cancer vaccines.

Poxviruses have been of great interest for many decades and are used as a vector to deliver a wide variety of heterologous genes. They are classified as double-stranded DNA viruses that contain a linear genome [74]. They can even carry genetic material as small as 24,000 bp. MVA is a subtype of poxvirus, which is derived from the vaccinia virus [69]. It is classified as an attenuated virus. As a result of several hundred passages behind chicken embryos, the virus was seriously weakened by deletion of about 30 kbp, which accounted for 15% of the entire genome. However, it exhibits adequate viral DNA replication and the ability to express the transgenic gene in host cells [79]. This replication and transcription of the virus takes place in the cytoplasm of host cells, which reduces the possibility of insertion mutagenesis [80]. There are several types of MVAs, which, interestingly, despite the same sequence of nucleotides within the coding regions, are characterized by different phenotypes. Among the advantages that promote MVA for use in the production of cancer vaccines are genetic stability, ease of manipulating the genome, and fairly high safety of use. In addition, it is characterized by inducing high immunogenicity, even when antiviral immunity has previously occurred [72]. MVA-BN has higher safety and a better ability to trigger an immune response than the other types [79]. It is noteworthy that MVA demonstrates a variety of tropisms to mammalian cells, in that infection occurs through passive membrane fusion [74]. The MVA-based vaccine is most promising for personalized therapy research [69]. The biggest downside seems to be the possibility of neurovirulence [74].

### 2.5. Dendritic Cell-Based Cancer Vaccine

**Figure 4:**
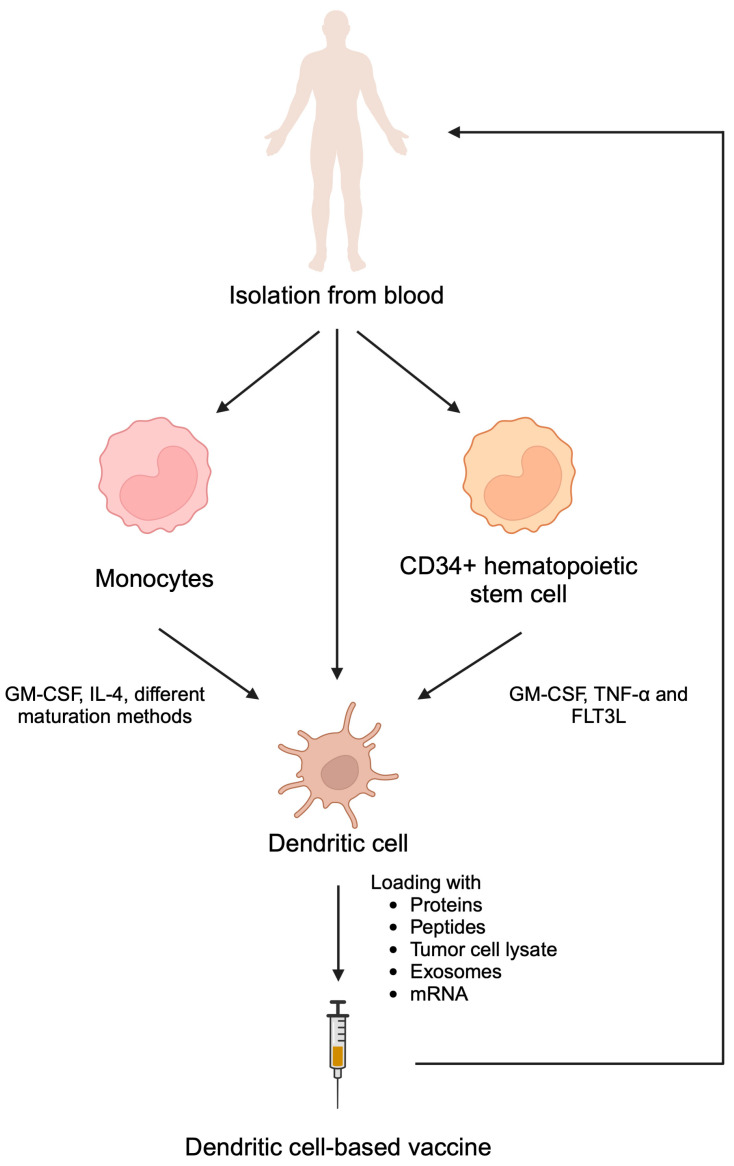
Dendritic cell-based vaccines can be prepared by isolating dendritic cell themselves as well as monocytes and CD34+ hematopoietic stem cells and inducing their differentiation towards dendritic cells. Subsequently, these cells can be stimulated with cancer antigens. Reintroduction of stimulated dendritic cells promotes cytotoxic immune reactions. Created in BioRender. Kiełbowski, K. (2025) https://BioRender.com/h52n415.

The concept of exploiting DCs for vaccine preparation arose after Ralph M. Steinman’s discovery of their central role in shaping the immune response [81]. DCs, the most potent members of the professional APC family, bridge the gap between the innate and adaptive immune response. After activation with an antigen, DCs gain the unique ability to migrate to lymph nodes, where they present the processed antigen to CD4^+^ and CD8^+^ T lymphocytes, thereby stimulating their activity [82,83]. This triggers both a humoral and cellular immune response, the power of which can potentially be harnessed to fight cancer. The overall framework behind their preparation is universal for all DC-based vaccines. First, DCs or their precursors are isolated from the patient’s blood and, if necessary, subjected to a maturation process. Then, the obtained DCs are activated with the use of antigens of varying sources and administered back to the patient. As a result, T cells are stimulated to mount a strong antitumor immune response that is responsible for the therapeutic effect of the vaccine. Preparation of DC vaccine is briefly summarized in Figure 4.

Since loading DCs with tumor antigens is a key step in vaccine preparation, the choice of a suitable form and source of the antigen is pivotal for the final product’s immune properties [84]. Due to the ease of preparation, short peptides classified as tumor-associated antigens are frequently used. Since they trigger a strong immune response, peptides can be presented on DCs only in a complex with a specific HLA subtype. The sine qua non condition for the effectiveness of the vaccine is therefore that the patient has the appropriate HLA subtype; otherwise, the immune response will be sparse [85,86]. For this reason, many clinical trials employing peptides which bind to HLA-A2 subtype excluded HLA-A2 negative patients, who constitute about 50% of the eligible population [87]. One method to overcome this problem is to use proteins as antigens instead of peptides. Indeed, after processing in DCs, proteins generate an array of peptides that can bind to multiple HLA subtypes [88]. Still, even vaccines that use proteins as antigens have mostly shown limited clinical benefit, probably due to the ability of the tumor to evade the immune response through an antigen loss mechanism [89].

An ingenious method that addresses this issue utilizes tumor lysate, a cocktail of the tumor’s DNA, RNA, proteins, and cell membrane fragments, along with a variety of signaling molecules, as the source of antigens. The cell lysate can be prepared either from autologous tumor cells (i.e., cells obtained from surgically resected tumor) [90,91,92] or an allogenic tumor cell line [93,94,95]. Generally, autologous tumor cell lysate is better suited for the patient as it contains a personal set of mutant antigens but is also difficult to obtain in large quantities [84]. Both autologous and allogenic cell lysates show promising clinical results and are used much more often than unlysed tumor cells [96]. Other forms of antigens applied in DC-based vaccine preparation include tumor exomes and mRNA. Exosomes are extracellular vesicles released by the cancer cells that contain tumor-associated proteins and mRNA [97]. Their advantage over autologous cell lysates is that they can be obtained directly from the patient’s blood or urine, without the need for tumor resection [98]. Similarly, mRNA can serve as a source of antigens that can be easily amplified and thus prepared in sufficient quantity even from a small tumor sample [99]. Despite relative safety, the practical use of mRNA is severely limited by its low stability [84].

The key milestone in the development of DC-based vaccines was achieved in 2010 with the FDA approval of the first therapeutic cancer vaccine, Sipuleucel-T, for metastatic castration-resistant prostate cancer [100]. According to the National Comprehensive Cancer Network (NCCN) guidelines, the agent is recommended for patients with asymptomatic or minimally symptomatic patients. Furthermore, there must not be liver metastases, while patients should be in ECOG 0-1 stage and have a life expectancy of more than 6 months [101]. Sipuleucel-T is manufactured from a mixture of mononuclear cells isolated via leukopheresis. After separation from the patient’s blood, the obtained cells are incubated with recombinant protein resulting from the fusion of prostatic acid phosphatase (PAP) with GM-CSF. This drives the activation of DCs, which are then, after purification, administered to the patient [102]. The entire process takes only about 36 to 48 h [103].

The clinical efficacy of Sipuleucel-T has been proven in numerous clinical trials. The landmark phase III IMPACT study enrolled 512 castration-resistant prostate cancer patients. It has shown that Sipuleucel-T therapy reduces the risk of death by 22% (*p* = 0.03) compared to placebo. This result translated into a 4.1-month improvement in the mean survival rate in the treatment group, with no significant impact on time to objective disease progression [104]. In subsequent STAMP and STRIDE trials, Sipuleucel-T was combined with abiratone acetate with prednisone and with enzalutamide, respectively. Recently long-term analyses demonstrated median OS of 33.3 months (STAMP trial) and 32.5 months (STRIDE trial) [105]. A large real-world study evaluating the combination of Sipuleucel-T with androgen receptor-targeting agents (ARTAs) confirmed the benefits of immunotherapy [106]. As several smaller studies have suggested, Sipuleucel-T might be safely combined with hormonotherapy [107], radiotherapy [108], and even other forms of immunotherapy [109,110], raising hopes for a more effective multi-target therapy for patients suffering from castration-resistant prostate cancer. Furthermore, researchers now focus on examining methods to improve the activity of Sipuleucel-T. Recently, Saeed et al. [111] proved that IL-15 enhances cytotoxic responses induced by the agents, thus paving the way for future research aiming to improve treatment efficacy. Similarly to the previously mentioned types of vaccines, several ongoing trials investigate the efficacy of Sipuleucel-T (NCT05806814 [112]; NCT06134232 [113]; NCT06100705 [114]; and NCT05751941 [115]). Interesting, NCT06134232 is going to investigate the efficacy of booster injection of the vaccine [113].

### 2.6. Other Dendritic Cell-Based Cancer Vaccines

DC-based cancer vaccines were investigated in conditions other than prostate cancer as well. For instance, a few recent reports described the use of DCs immunotherapy in lung cancer, one of the most common types of malignancies associated with the highest cancer-related death rate [116]. Ding et al. [117] described the use of autologous DCs pulsed with neoantigens in a phase 2 clinical trial including patients with relapsed advanced lung cancer. Twelve patients were administered with the vaccine, which proved to be safe, as no grade 3–4 AEs were present. All patients experienced grade 1–2 local skin injection site reactions while grade 2 neutropenia and rash were observed in two separate patients. The median PFS and OS were 5.5 and 7.9 months, respectively, while 25% of patients achieved an objective response rate. Thus, the use of neoantigen pulsed DC treatment emerges as a promising treatment in patients with recurrent advanced disease [117]. Ingels et al. [118] published a result of the phase I trial in which the authors engineered DCs harboring the mRNA of neoantigens. Similarly to the previous trial, the vaccine was safe, while AEs were self-limiting in all six of the included patients. Regarding the efficacy, three patients experienced disease progression, while the other three patients were free of relapse. Thus, these highly advanced vaccines could potentially represent a novel treatment method in patients with recurrent advanced disease who were previously treated with several other lines of therapy. The manageable safety profile of this cellular treatment is highly promising.

Nevertheless, the use of DC-based vaccines was not only examined in advanced disease, but also to prevent recurrence. Recently, van’t Land and colleagues [119] published a combined phase I/II clinical trial that examined DC immunotherapy as an adjuvant treatment in patients after surgical treatment of pancreatic cancer. The primary end point analysis was performed in 38 patients, thus demonstrating a significantly larger population than in the previous trials involving lung cancer patients. A grade 3 AE was observed only in one patient. The estimated 2-year OS and recurrence-free survival were 83% and 64%, respectively. Interestingly, another method to utilize DC-based vaccine is to use DC-derived extracellular vesicles, such as microvesicles or exosomes, with initial studies showing increased antitumor immune responses [120,121]. Table 1 summarizes clinical studies investigating safety and efficacy of discussed anticancer vaccines.

## 3. Conclusions and Future Perspectives

To conclude, anticancer vaccines represent an evolving field of immunotherapy, with a significant number of strategies already developed. Importantly, anticancer vaccines offer a direct targeting of tumor cells, thus contributing to the emerging area of personalized oncology. A recently published case report by Kosumi et al. [122] demonstrated the involvement of anticancer vaccines in individualized treatment. Researchers described an NSCLC patient that was treated with erlotinib (anti-EGFR therapeutic) and with DCs pulsed with a Wilms’ tumor 1 and MUC1 vaccine. Therefore, this treatment strategy involved targeted therapy and immunotherapy, thus reducing the tumor size by over 60% and continuing the treatment for 699 days. Furthermore, one needs to mention the benefits of HPV vaccines. With a variety of HPV-related cancers, the use of HPV vaccines showed an important role in cancer prevention. However, the use of the HPV vaccine recently showed clinical benefits in patients with HPV-associated head and neck squamous cell cancer [123]. Moreover, more vaccines targeting HPV-related malignancies are being developed with the aim of cancer treatment [124,125].

Early studies demonstrated the benefits of targeting prostate cancer with anticancer vaccines, which led to the approval of the Sipuleucel-T vaccine. Sipuleucel-T and DNA-based vaccines utilize PAP to target prostate cancer cells. However, in case of other malignancies, it is required to analyze tumor samples to determine underlying driving mechanisms, mutations, and overexpressed molecules. Vaccines that can encode or induce the targeting of multiple antigens offer promising efficacy, especially in malignancies with high intra-tumor heterogeneity. Liquid biopsy could perhaps be used to develop targeted anticancer vaccines in the future. Recent studies demonstrated that anticancer vaccines demonstrate increased benefits when co-administered with other immunotherapeutics. However, researchers investigated the potential benefits of anticancer therapeutic cancer vaccines in monotherapy or in cancers less sensitive to classic immunotherapy. The benefits of vaccines as single agents and their combination with chemotherapy, immunotherapy, and targeted therapy is an important area that should be explored further. Performed clinical trials showed that anticancer vaccines could prolong the metastasis-free period, which should translate into a significantly higher quality of life for the affected patients.

## Figures and Tables

**Table 1:** Summary of selected clinical studies involving anticancer therapeutic vaccines.

Vaccine	Type of Vaccine	Study	Population	Main Outcomes	Reference
mRNA4157 (V940)	mRNA	KEYNOTE-942	Melanoma	RFS at 18 months: Study group: 79% Control group: 62%	[18]
mRNA-4359	mRNA	NCT05533697	Advanced solid tumors	50% of patients achieved stable disease	[31]
Autogene-cevumeran	mRNA	Study by Rojas et al.	Pancreatic ductal adenocarcinoma	During 18 months of follow-up, RFS and OS	[34]
pTVG-hp	Plasmid DNA vaccine	Study by Tonelli et al.	Non-metastatic prostate cancer	OS in castration sensitive patients: 12.3 years OS in castration-resistant patients: 4.5 years	[55]
pTVG-AR	Plasmid DNA vaccine	Study by Kyriakopoulos et al.	Prostate cancer	Study showed that the agent is safe and can induce anticancer responses	[59]
ERBB2 plasmid vaccine	Plasmid DNA vaccine	Study by Disis et al.	Breast cancer	Study showed that the agent is safe and enhance the presence of memory T cells	[61]
GNOS-PV02	Plasmid DNA vaccine	Study by Yarchoan et al.	Hepatocellular carcinoma	When combined with pembrolizumab, the ORR was approximately 30%	[63]
NEO-PV-01	Neoantigen vaccine	NCT02897765	NSCLC, melanoma, bladder cancer	Study showed that the agent combined with anti-PD-1 therapy is safe and can induce anticancer responses	[65]
		Study by Awad et al.	metastatic non-squamous NSCLC	Combination of the vaccine with classic chemotherapeutics was well tolerated	[66]

RFS—recurrence free survival; OS—overall survival; ORR—overall response rate.

## Data Availability

Not applicable.

## Abbreviations

The following abbreviations are used in this manuscript:

NAV	Nucleic acid-based vaccines
TAA	Tumor-associated antigens
TSA	Tumor specific antigens
CDC	Complement-mediated cytotoxicity
ADCC	Antibody-dependent cellular cytotoxicity
APC	Antigen presenting cell
DC	Dendritic cell
HLA	Human leukocyte antigen
NCCN	National Comprehensive Cancer Network
VNTR	Variable number of tandem repeats
MUC1	Mucin 1
TIL	Tumor infiltrating lymphocytes
PAMPs	Pathogen-associated molecular patterns
PAP	Prostatic acid phosphatase
PSA	Prostate-specific antigen
PSMA	Prostate-specific membrane antigen
PSCA	Prostate stem cell antigen
PFS	Progression-free survival
OS	Overall survival
RFS	Recurrence free survival
AE	Adverse event
TEAE	Treatment-emergent adverse events
